# *Ramulus Mori* (Sangzhi) Alkaloids Improve Pancreatic β-Cell Function Through Gut Microbial and Intra-Islet 2-Methoxyestradiol Biosynthesis

**DOI:** 10.3390/biomedicines13082013

**Published:** 2025-08-19

**Authors:** Nan Wu, Lusi Lu, Yiming Liu, Sunyue He, Chunyi Xu, Ying Wu, Yuchen Zhao, Xihua Lin, Wenjing Zhang, Jiaqiang Zhou

**Affiliations:** Department of Endocrinology and Metabolism, Sir Run Run Shaw Hospital, Zhejiang University School of Medicine, Hangzhou 310058, China; wunan1105@163.com (N.W.); 3316095@zju.edu.cn (L.L.); lymqd2020@163.com (Y.L.); hesunyue@163.com (S.H.); xuchunyi227@163.com (C.X.); wuying2013@126.com (Y.W.); zjuzyc1002@163.com (Y.Z.); linxihua@zju.edu.cn (X.L.)

**Keywords:** *Ramulus Mori* (Sangzhi) alkaloids, gut microbiota, metabolites, β-cell function, 2-methoxyestradiol

## Abstract

**Background**: *Ramulus Mori* (Sangzhi) Alkaloids (SZ-A) are natural hypoglycemic compounds known to enhance insulin secretion. Given the emerging role of the gut microbiota in regulating β-cell function, in this study, we aimed to investigate whether SZ-A exert their beneficial effects through modulating the gut microbiota and its metabolites. **Methods**: A diabetic mouse model was established using a high-fat diet and streptozotocin, followed by 20 weeks of SZ-A treatment. Gut microbiota and metabolites were profiled via 16S rRNA sequencing and liquid chromatography–mass spectrometry, respectively. Spearman’s correlation analysis was used to explore associations between gut microbiota and metabolites. Single-cell RNA sequencing (scRNA-seq) was used to assess gene expression and signaling pathway changes in β cells. **Results**: Our results demonstrate that SZ-A alleviated hyperglycemia and increased islet numbers in T2DM mice. SZ-A treatment also reshaped the gut microbiota, notably enriching quantities of *Lactobacillus* and *norank_f__Eubacterium_coprostanoligenes_group*, which may contribute to increasing levels of 2-methoxyestradiol (2-ME), a bioactive metabolite. Moreover, scRNA-seq revealed an increased proportion of COMT^+^ cells in the islets, suggesting that 2-ME may also be synthesized within the islets. In vitro, 2-ME suppressed HIF-1α signaling and promoted insulin secretion, indicating that 2-ME may act as a crucial mediator of the beneficial effects of SZ-A. **Conclusions**: SZ-A improve β-cell function by increasing 2-ME levels via gut microbiota modulation and islet production, ultimately suppressing HIF-1α signaling and restoring β-cell homeostasis.

## 1. Introduction

Diabetes mellitus is a major global health challenge. In 2024, the International Diabetes Federation estimated that there were 589 million adults worldwide living with diabetes, the majority of whom had type 2 diabetes mellitus (T2DM). This number is projected to rise to approximately 700 million by 2045 [1,2]. Insulin resistance and β-cell dysfunction both contribute to the progression of T2DM [3,4,5]. Therefore, preserving β-cell mass and function is essential for glycemic control. Emerging evidence shows that the gut microbiota is a critical regulator of metabolic homeostasis, capable of influencing β-cell physiology through its metabolites. For instance, dietary choline is metabolized by gut bacteria to produce trimethylamine N-oxide, contributing to β-cell dysfunction [6]. Intestinal bacterial ligands of nucleotide-binding oligomerization domain containing 1 directly regulate insulin trafficking in β-cells [7]. Moreover, fecal microbiota transplants from lean individuals have enhanced insulin sensitivity in T2DM patients [8]. Collectively, these findings highlight the pivotal role of the gut microbiota in β-cell regulation and suggest novel microbiota-targeted strategies for diabetes therapy.

Natural products are increasingly valued for their therapeutic efficacy and low toxicity [9]. *Ramulus Mori* (Sangzhi) Alkaloids (SZ-A), a group of polyhydroxy alkaloids derived from mulberry branches, exhibit glucose-level-lowering effects comparable to those of acarbose in T2DM patients, primarily through inhibiting intestinal α-glucosidase [10]. In addition to reducing HbA1c levels, SZ-A also improve lipid profiles and help reduce weight [11]. Notably, in HFD-induced obese mice, SZ-A alleviate metabolic disorders by modulating the gut microbiota, particularly with respect to the Firmicutes/Bacteroidota ratio [12], and enhance insulin secretion by preventing β-cell dedifferentiation and apoptosis [13]. These findings suggest a potential link between SZ-A’ modulation of gut microbiota modulation and protection of β-cells. However, the precise mechanisms underlying this effect are not yet fully understood. To investigate this, we performed microbiome sequencing of cecal contents, conducted cecal metabolomics, and performed single-cell RNA sequencing (scRNA-seq) of pancreatic islets to identify crucial microbial and metabolic factors involved in SZ-A-mediated β-cell protection.

In this study, we demonstrate that SZ-A improve β-cell function and glycemic control through a dual mechanism: microbiota-dependent elevation of 2-methoxyestradiol (2-ME) and intra-islet biosynthesis by COMT^+^ subpopulations. Mechanistically, 2-ME suppresses hypoxia-inducible factor-1α (HIF-1α) and its downstream glycolytic regulators (LDHA, PDK1, and PGK1), thereby ameliorating β-cell dysfunction under diabetic stress conditions. These findings highlight a previously unrecognized microbial–islet crosstalk mediated by 2-ME and reveal SZ-A’s therapeutic potential in regard to diabetes.

## 2. Materials and Methods

### 2.1. Materials

SZ-A powder (Lot No.: J202310021) was provided by the Department of Research & Development of Beijing Wehand-Bio Pharmaceutical Co., Ltd. (Beijing, China). The primary alkaloid components of the SZ-A include 1-deoxynojirimycin (DNJ); 1,4-dideoxy-1,4-imino-D-arabinitol (DAB); and fagomine (FAG). The chemical structures of these compounds are shown as Supplementary Materials (Figure S1).

### 2.2. Animals

All animal procedures were approved by the Animal Ethical and Welfare Committee of Zhejiang Chinese Medical University (ethics approval number: IACUC-202306-09) and conducted in accordance with relevant ethical guidelines. Eight-week-old male C57BL/6J mice were housed under specific-pathogen-free conditions (22 ± 2 °C, 55% ± 5% relative humidity, and a 12 h light/dark cycle). After one week of acclimatization, the mice were randomly assigned using a computer-generated randomization sequence to either a control group fed a standard chow diet or an experimental group fed a high-fat diet (HFD; 60% fat, 20% carbohydrate, and 20% protein; D12492, Research Diets Inc., New Brunswick, NJ, USA). After four weeks of adherence to the HFD, the mice received intraperitoneal injections of streptozotocin (STZ; 30 mg/kg; S8050; Solarbio Life Science, Beijing, China) for three consecutive days. Diabetes was defined as a fasting blood glucose level ≥ 11.1 mmol/L in two separate measurements. The groups included a saline-treated control group (*n* = 10), a diabetic model (DM) group (*n* = 8), and three SZ-A treatment groups (*n* = 8 each) that received SZ-A at doses of 100, 150, or 200 mg/kg, administered once daily via oral gavage for 20 weeks. Random blood glucose levels were monitored throughout the experiment. At the end of the study, the mice were fasted overnight before being euthanized. Fecal samples from the cecum and fasting serum samples were obtained for subsequent analysis.

### 2.3. Oral Glucose Tolerance Test (OGTT) and Insulin Tolerance Test (ITT) Assays

In the OGTT, the mice were fasted overnight for 16 h and gavaged with a 1 g/kg glucose solution. In the ITT, the mice were fasted for 4 h and administered an intraperitoneal injection of 1 U/kg of insulin. Blood glucose levels were measured at various points after the injection.

### 2.4. Hematoxylin and Eosin (H&E) Staining

H&E staining was performed as previously described [14]. Briefly, pancreatic tissue samples were fixed in 4% paraformaldehyde and then dehydrated using graded ethanol solutions. The tissues were then embedded in paraffin. Subsequently, we acquired 4 μm thick sections of the tissues and subjected them to H&E staining for histological evaluation.

### 2.5. Analysis of Gut Microbiota Composition

In accordance with the manufacturer’s protocols, DNA was extracted from the cecal contents of the mice. Primers specific to the V3-V4 hypervariable regions of the 16S rRNA gene were designed for PCR amplification. Sequencing was performed using the PE300 platform (Illumina, San Diego, CA, USA), and data analysis was executed on the MajorBio Cloud Platform (MajorBio, Shanghai, China). We used the mothur software (version 1.30.2) to calculate alpha diversity indices such as the ACE, Sobs, Shannon, and Chao indices. Principal Component Analysis (PCA) with Bray–Curtis distances was performed to compare microbial community structures among samples.

### 2.6. Non-Targeted Metabolomic Analysis

Metabonomic analysis of cecal contents was performed utilizing an ultra-high-performance liquid chromatography–quadrupole (UHPLC-Q) Exactive HF-X system (Thermo Fisher Scientific, Waltham, MA, USA). Samples were separated on a Hollow Structural Sections (HSS) T3 column (100 × 2.1 mm, 1.8 µm) and analyzed through mass spectrometry (MS). Mobile phase A consisted of 95% water and 5% acetonitrile with 0.1% formic acid, while mobile phase B was composed of 47.5% acetonitrile, 47.5% isopropanol, and 5% water with 0.1% formic acid. Mass-spectrometric data were acquired in both positive and negative ion modes. The sheath gas flow rate was set to 50 psi, the spray voltage was 3500 V, and the ion transfer tube temperature was maintained at 325 °C. The raw LC-MS data were preprocessed using the Progenesis QI software v3.0 (Waters Corporation, Milford, MA, USA) and further analyzed via the MajorBio cloud platform. PCA and Partial Least Squares Discriminant Analysis (PLS-DA) were conducted through the R package ropls (Version 1.6.2). Metabolites with a Variable Importance in Projection (VIP) score greater than 1 and a *p*-value under 0.05 were considered significantly differentially abundant. The biochemical pathways of these metabolites were further analyzed using the Kyoto Encyclopedia of Genes and Genomes (KEGG) database, allowing for pathway-based classification.

### 2.7. Islet Dissection

Mouse islets were isolated using intraductal digestion with Collagenase type V (C9263; Sigma-Aldrich, St. Louis, MO, USA), as previously described [15]. Subsequently, the islets underwent refinement via centrifugation through a Histopaque 1077 (10771; Sigma-Aldrich) density gradient. Purified islets were selected under a stereo microscope. Islets were cultured in Roswell Park Memorial Institute (RPMI)-1640 medium (C11875500BT; Gibco, Grand Island, NY, USA), supplemented with 10% fetal bovine serum (FBS; SE100-011; Vistech, New Zealand), 100 μg/mL of streptomycin, and 100 U/mL of penicillin.

### 2.8. Single-Cell Suspension

The islets were enzymatically dissociated using TrypLETM Express Enzyme (12604-013; Gibco) at 37 °C for 15 min [14]. The reaction was stopped by adding 2 mmol/L of EDTA in phosphate-buffered saline (PBS). The cells were subsequently resuspended in 2.5 mmol/L of EDTA and 0.5% bovine serum albumin (BSA) in PBS. Cell counts and cell viability were assessed using the BD Rhapsody scRNA-seq platform (BD Biosciences, San Jose, CA, USA).

### 2.9. ScRNA-Seq and Data Analysis

Single cells obtained from the islets were prepared for scRNA-seq using the BD Rhapsody system [16]. First, the cell suspension was diluted and loaded into a microwell cartridge. Once the cells were captured, beads coated with oligonucleotides were introduced to bind mRNA from each cell. These beads were magnetically harvested and pooled for reverse transcription; then, cDNA amplification and library preparation were carried out. The libraries were subjected to high-throughput transcriptomic sequencing using the NovaSeq 6000 platform (Illumina). The raw gene expression matrix was transformed into a Seurat object using the Seurat R package (Version 5.0.1). Cells were filtered out if they had fewer than 200 or more than 6500 detected genes or if mitochondrial genes constituted over 25% of their total gene count. The R Harmony package (Version 1.2.0) was applied for batch correction. Cell clusters were visualized utilizing the uniform manifold approximation and projection (UMAP) technique. Gene expression changes were further analyzed and presented using the VlnPlot and DotPlot functions in Seurat.

### 2.10. Cell Culture

The mouse insulinoma cell line, β-TC-6, was cultured in DMEM medium supplemented with 15% FBS, 100 U/mL of penicillin and 100 µg/mL of streptomycin. The cells were maintained at 37 °C in a humidified atmosphere with 5% CO_2_.

### 2.11. Cell Viability Assay

The β-TC-6 cells were exposed to a range of concentrations of SZ-A (12.5, 25, 50, 100 μg/mL) for 24 h. Thereafter, the cells were incubated for 1.5 h at 37 °C in the dark with a culture medium containing the Cell Counting Kit-8 (CCK-8; 40203ES60; Yeasen Biotech, Shanghai, China) solution, as per the manufacturer’s instructions. Absorbance at 450 nm was then measured.

### 2.12. Western Blotting

Western blotting was performed as previously described [17]. Briefly, the islets were treated with cell lysis buffer (FD008; FdBio Science, Hangzhou, China) supplemented with a protease inhibitor cocktail (FD1001; FdBio Science). Total protein extracts were denatured, separated via gel electrophoresis, and transferred to PVDF membranes. After blocking, the membranes were incubated overnight at 4 °C with the following primary antibodies: anti-catechol-O-methyltransferase (COMT, 1:1000; A23600; Abclonal, Wuhan, China), anti-HIF-1α (1:500; NB100-105; NOVUS, St. Louis, MO, USA), anti-lactate dehydrogenase A (LDHA, 1:1000; A21893; Abclonal), anti-pyruvate dehydrogenase kinase 1 (PDK1, 1:1000; A24749; Abclonal), anti-phosphoglycerate kinase 1 (PGK1, 1:1000; A12686; Abclonal), anti-glyceraldehyde 3-phosphate dehydrogenase (GAPDH, 1:10,000; AC001; Abclonal), and anti-heat shock protein 90 (HSP90, 1:2000; A5027; Abclonal). Subsequently, the membranes were incubated with labeled anti-mouse or anti-rabbit secondary antibodies (1:5000; AS003 and AS014; Abclonal). The immunoreactive protein bands were visualized using a chemiluminescence kit (FD8030; FdBio Science).

### 2.13. 2-ME Measurements

Serum and islet concentrations of 2-ME were detected using a mouse 2-ME enzyme-linked immunosorbent assay (ELISA) kit (LCSJZF23854; LunChangShuoBiotech, Xiamen, China). Briefly, samples were combined with enzyme-linked antibodies in the wells and incubated at 37 °C for 1 h. After a 15 min incubation with substrate solutions in the dark at 37 °C, stop solution was added, and the absorbance at 450 nm was measured. The islet 2-ME levels were normalized to the protein concentration.

### 2.14. Glucose-Stimulated Insulin Secretion (GSIS)

The GSIS assay was conducted as previously described [18]. β-TC-6 cells were exposed to 500 μmol/L of PA and various concentrations of SZ-A (12.5, 25, 50, and 100 μg/mL) for 24 h. The cells were preincubated in Krebs–Ringer bicarbonate HEPES (KRBH) buffer supplemented with 0.1% BSA for 30 min. Subsequently, the cells were incubated for 1 h in KRBH buffer containing either 2.8 mmol/L or 16.7 mmol/L of glucose. The insulin levels in the supernatant were measured using an Insulin ELISA kit (MS300; Ezassay, Shenzhen, China), and the results were normalized to the protein concentrations.

The islets were cultured in a medium containing 500 nmol/L of 2-ME for 24 h, as described in a previous study [19], and then subjected to an additional 24 h incubation with 500 μmol/L of PA. After being incubated in KRBH buffer with 0.1% BSA for 30 min, the islets were sequentially exposed to 2.8 mmol/L and 16.7 mmol/L glucose in KRBH buffer for 1 h. The insulin levels in the supernatant were measured using the same procedure used for the β-TC-6 cells.

### 2.15. Statistical Analysis

The data are presented as means ± SEM. Statistical analyses were performed using GraphPad Prism version 10.2.3. Data normality was assessed prior to hypothesis testing. For normally distributed datasets, one-way ANOVA followed by Dunnett’s post hoc test for multiple comparisons was used. For non-normally distributed data, the Kruskal–Wallis test was applied, followed by Dunn’s post hoc test. Differential gene expression between two groups in the scRNA-seq data was evaluated using the Wilcoxon rank-sum test. Correlations between gut microbiota and metabolite profiles were assessed using Spearman’s rank correlation analysis. A *p*-value < 0.05 was considered statistically significant.

## 3. Results

### 3.1. SZ-A Improve Glucose Metabolism and Protect β-Cell Function in Diabetic Mice

**Figure 1:**
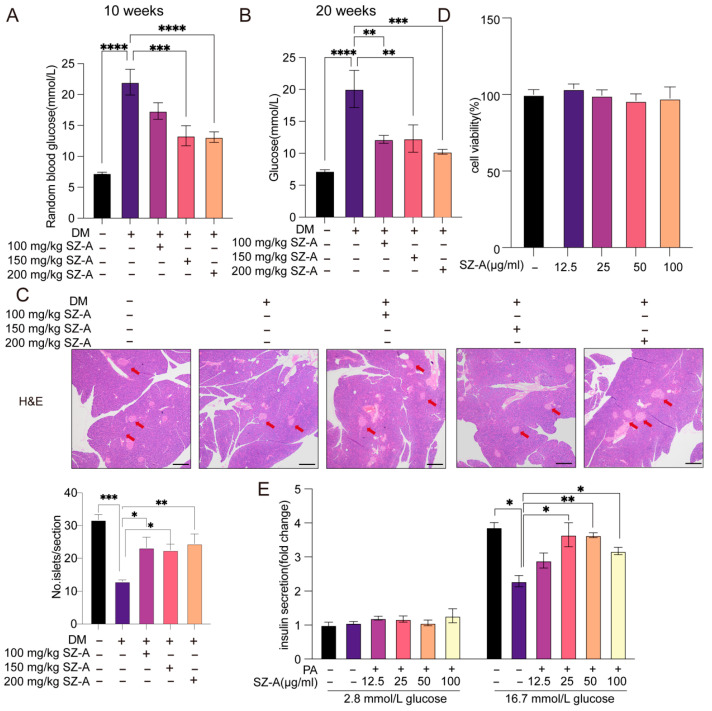
SZ-A improve glucose metabolism and protect β-cell function in diabetic mice. (**A**) Random blood glucose levels at 10 weeks in control (CTR), diabetic mice (DM), and SZ-A-treated diabetic mice at varying concentrations (*n* = 8–10). (**B**) Random blood glucose levels at 20 weeks in CTR, DM, and SZ-A-treated diabetic mice at varying concentrations (*n* = 8–10). (**C**) H&E staining of pancreas sections from CTR, DM, and SZ-A-treated diabetic mice, along with the count of islets per pancreas section (*n* = 4). Scale bar, 500 µm. The red arrow indicates the islet. (**D**) Cell viability was measured using the CCK-8 assay in β-TC-6 cells treated with varying concentrations of SZ-A (*n* = 5). (**E**) GSIS in β-TC-6 cells treated with CTR, 500 µmol/L PA and PA combined with varying concentrations of SZ-A (*n* = 3). *, *p* < 0.05, ** *p* < 0.01, ***, *p* < 0.001, ****, *p* < 0.0001.

The HFD/STZ-induced diabetic mice exhibited significantly higher random blood glucose levels than the normal controls (*p* < 0.05). Following SZ-A treatment, blood glucose levels were markedly reduced across different concentrations (100, 150, and 200 mg/kg), demonstrating a potent antihyperglycemic effect (Figure 1A,B). Among these concentrations, 150 mg/kg was chosen for subsequent in vivo studies because it induces a faster onset of effects and exhibits robust efficacy at a relatively low dose. H&E staining and quantitative analysis of islets revealed a substantial increase in islet quantities in the SZ-A-treated mice in comparison to the untreated diabetic mice (*p* < 0.05), indicating a potential role of SZ-A in β-cell preservation or regeneration (Figure 1C).

β-TC-6 cells were used to further investigate the effects of SZ-A on β-cell function. Cell viability across a range of SZ-A concentrations (12.5, 25, 50, and 100 μg/mL) was assessed using a CCK-8 assay, confirming the absence of cytotoxicity (Figure 1D). To assess the effect of SZ-A on insulin secretion, GSIS assays were performed on the PA-treated β-TC-6 cells under low- and high-glucose conditions. PA significantly impaired insulin secretion under the high-glucose conditions; this function was notably restored by SZ-A treatment at concentrations of 25 μg/mL and above (*p* < 0.05). No significant differences were observed under low-glucose conditions (Figure 1E). These results suggest that SZ-A ameliorate PA-induced β-cell dysfunction.

### 3.2. SZ-A Modulate the Composition of Gut Microbiota

**Figure 2:**
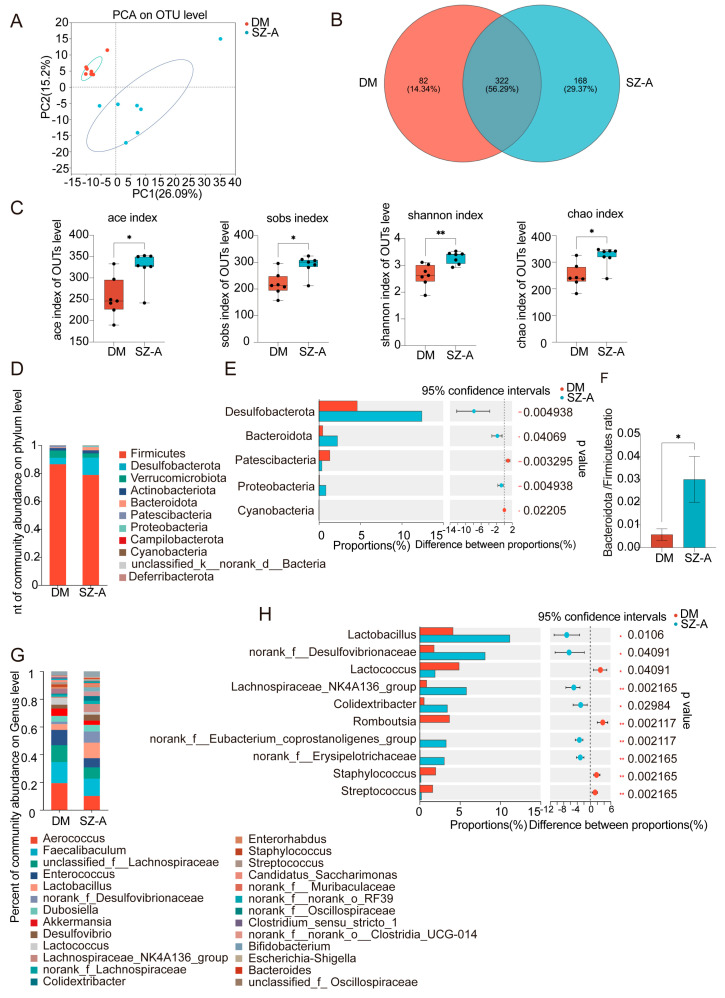
SZ-A modulate the composition of the gut microbiota. (**A**) PCA plots of gut microbiota profiles in DM and those treated with 150 mg/kg SZ-A (*n* = 7). (**B**) Venn diagrams of the OTUs of gut microbiota identified in DM and those treated with SZ-A (*n* = 7). (**C**) ACE, Sobs, Shannon, and Chao indexes to describe the alpha diversity of gut microbial communities (*n* = 7). (**D**) The relative phylum-level abundance of gut microbiota in DM and those treated with SZ-A (*n* = 7). (**E**) The phylum-level alterations to gut microbiota in DM and those treated with SZ-A (*n* = 7). (**F**) Bacteroidota/Firmicutes ratio in DM and those treated with SZ-A (*n* = 7). (**G**) The relative genus-level abundance of gut microbiota in DM and those treated with SZ-A (*n* = 7). (**H**) The genus-level alterations to gut microbiota in DM and those treated with SZ-A (*n* = 7). *, *p* < 0.05, ** *p* < 0.01.

Given the well-established link between diabetes and gut microbiota dysbiosis, we investigated the effects of SZ-A on gut microbial composition and diversity. PCA of the fecal samples demonstrated clear separation between the diabetic mice and those treated with 150 mg/kg of SZ-A, indicating modulation of the gut microbiota (Figure 2A). Venn diagram analysis revealed an increase in OTU numbers following SZ-A treatment, reflecting enhanced microbial richness (Figure 2B). Furthermore, the diversity indices employed (ACE, Sobs, Shannon, and Chao) indicated there was a significant increase in microbial diversity in the SZ-A-treated mice in comparison to the diabetic controls (*p* < 0.05) (Figure 2C). These results suggest that SZ-A positively regulate gut microbiota, which may contribute to their metabolic benefits.

To further characterize microbial alterations, we analyzed taxonomic composition at the phylum level. SZ-A treatment significantly increased the relative abundance of *Desulfobacterota* and *Bacteroidota* while also reducing the levels of *Patescibacteria* and *Cyanobacteria* (*p* < 0.05) (Figure 2D,E). Notably, SZ-A restored the *Bacteroidota*-to-*Firmicutes* ratio, which was typically lower in the diabetic mice, suggesting a correction of gut microbiota imbalance (*p* < 0.05) (Figure 2F). At the genus level, SZ-A markedly reduced quantities of potentially harmful bacteria such as *Romboutsia*, *Staphylococcus*, and *Streptococcus* and enriched quantities of beneficial taxa including *Lactobacillus*, *Lachnospiraceae_NK4A136_group*, *Colidextribacter*, *norank_f__Oscillospiraceae*, and *norank_f__Eubacterium_coprostanoligenes_group* (*p* < 0.05, Figure 2G,H). Collectively, these findings indicate that SZ-A treatment significantly reshapes the gut microbiota in diabetic mice, as evidenced by increased microbial diversity, restoration of microbial balance, and enrichment of beneficial bacterial genera.

### 3.3. SZ-A Modulate Gut Lipid Metabolism in Diabetic Mice

**Figure 3:**
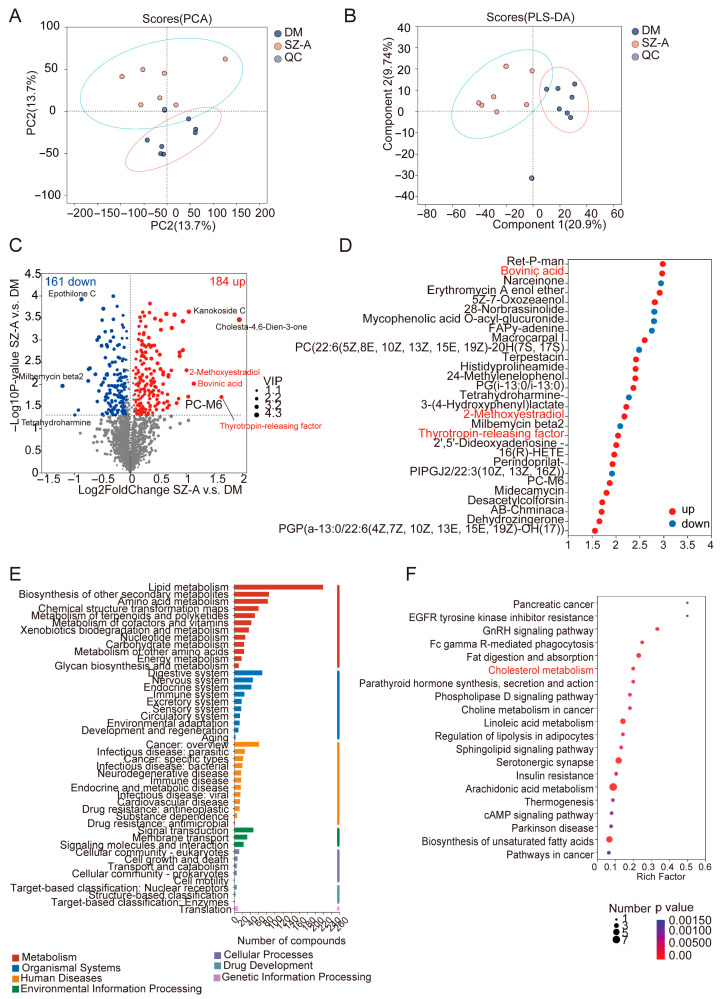
SZ-A modulate gut lipid metabolism in diabetic mice. (**A**) PCA and (**B**) PLS-DA plots of fecal metabolomic profiles in DM and those treated with SZ-A (*n* = 7). (**C**) Volcano plots of differential fecal metabolites in DM and those treated with SZ-A (*n* = 7). (**D**) Top 30 differential fecal metabolites ranked by VIP scores in DM and those treated with SZ-A (*n* = 7). (**E**) KEGG pathway classification and (**F**) KEGG pathway enrichment analysis of the differential fecal metabolites in DM and those treated with SZ-A (*n* = 7).

Furthermore, we conducted comprehensive fecal metabolomic profiling to assess the impact of SZ-A on gut metabolites. PCA and PLS-DA revealed a clear metabolic separation between the diabetic and SZ-A-treated mice, indicating that the SZ-A profoundly altered the gut metabolome (Figure 3A,B). VIP analysis identified a total of 345 differential metabolites, with 184 upregulated and 161 downregulated after SZ-A treatment (Figure 3C). Among these, the levels of bovinic acid, thyrotropin-releasing factor, and 2-ME were significantly increased in the SZ-A-treated mice in comparison to the diabetic controls (Figure 3C,D).

KEGG pathway analysis revealed that the SZ-A primarily influenced lipid metabolism (Figure 3E), inducing significant enrichment in cholesterol metabolism, biosynthesis of unsaturated fatty acids, fat digestion and absorption, and linoleic acid metabolism (Figure 3F). Notably, among these lipid-related pathways, cholesterol metabolism was one of the most significantly affected, suggesting that modulation of cholesterol homeostasis may be a key mechanism underlying the metabolic benefits of SZ-A.

### 3.4. SZ-A Enhance 2-ME Production by Modulating Specific Gut Microbiota

**Figure 4:**
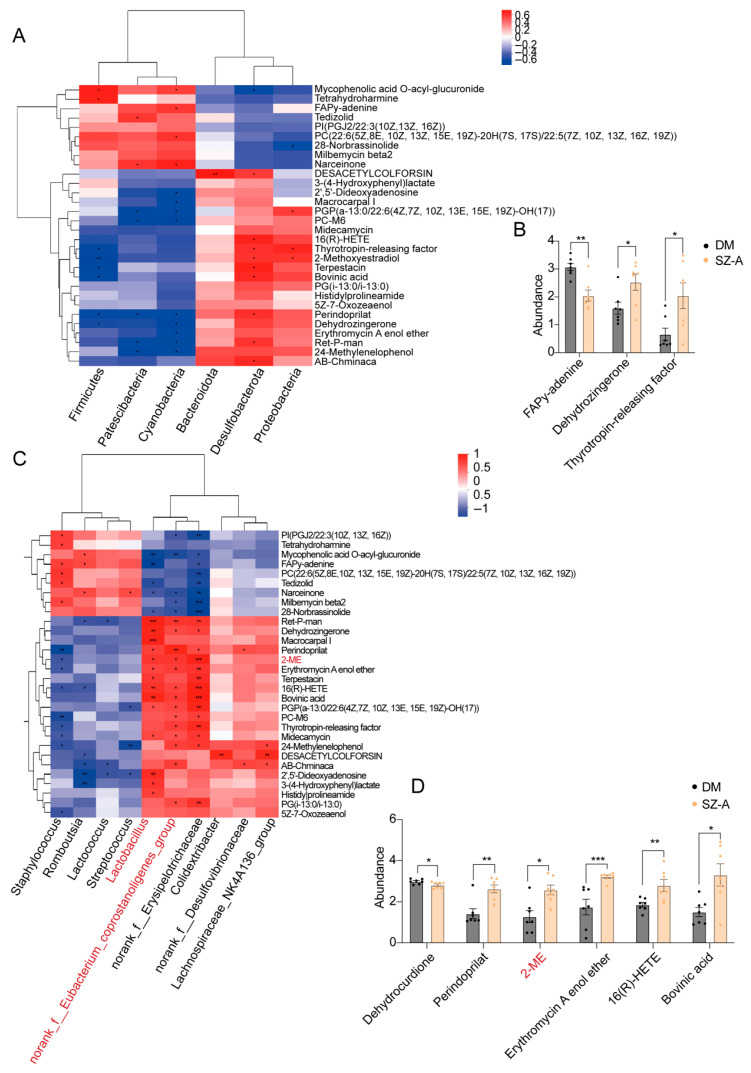
SZ-A enhance 2-ME production by modulating specific gut microbiota. (**A**) Spearman’s correlation analysis between the top 30 differential fecal metabolites and the gut microbiota at the phylum level in DM and those treated with SZ-A (*n* = 7). (**B**) The abundance of the metabolites FAPy-adenine, Dehydrozingerone, and thyrotropin-releasing factor in DM and those treated with SZ-A (*n* = 7). (**C**) Spearman’s correlation analysis between the top 30 differential fecal metabolites and the gut microbiota at the genus level in DM and those treated with SZ-A (*n* = 7). (**D**) The abundance of the metabolites Dehydrocurdione, Perindoprilat, 2-ME, Erythromycin A enol ether, 16(R)-HETE, and bovinic acid in DM and those treated with SZ-A (*n* = 7). *, *p* < 0.05, ** *p* < 0.01, *** *p* < 0.001.

A Spearman correlation analysis was performed to investigate the associations between fecal metabolites and gut microbiota. At the phylum level, *Cyanobacteria* showed a positive correlation with FAPy-adenine, a marker of oxidative DNA damage [20], and a negative correlation with the antioxidant Dehydrozingerone [21]. Similarly, *Firmicutes* exhibited a negative correlation with Dehydrozingerone. Thyrotropin-releasing factor, which plays a role in metabolism and insulin sensitivity [22], was negatively correlated with *Firmicutes* but positively correlated with *Desulfobacterota* and *Proteobacteria* (Figure 4A,B).

At the genus level, *norank_f__Erysipelotrichaceae*, *Lactobacillus*, and *norank_f__Eubacterium_coprostanoligenes_group* were significantly correlated with at least 16 fecal metabolites, many of which are involved in gut lipid metabolism (Figure 4B). Notably, *Lactobacillus* and *norank_f__Eubacterium_coprostanoligenes_group* have previously been reported to participate in microbial cholesterol metabolism [23,24]. One of the most significantly altered metabolites identified was 2-ME, a cholesterol-derived compound known to regulate glucose homeostasis [25]. Spearman’s correlation analysis revealed a strong positive association between the abundance of *Lactobacillus*/*norank_f__Eubacterium_coprostanoligenes_group* and fecal levels of 2-ME (Figure 4C,D). These results suggest that microbial changes induced by SZ-A may promote the biosynthesis of 2-ME.

### 3.5. SZ-A Elevate 2-ME Levels by Enhancing COMT Expression in Islets

**Figure 5:**
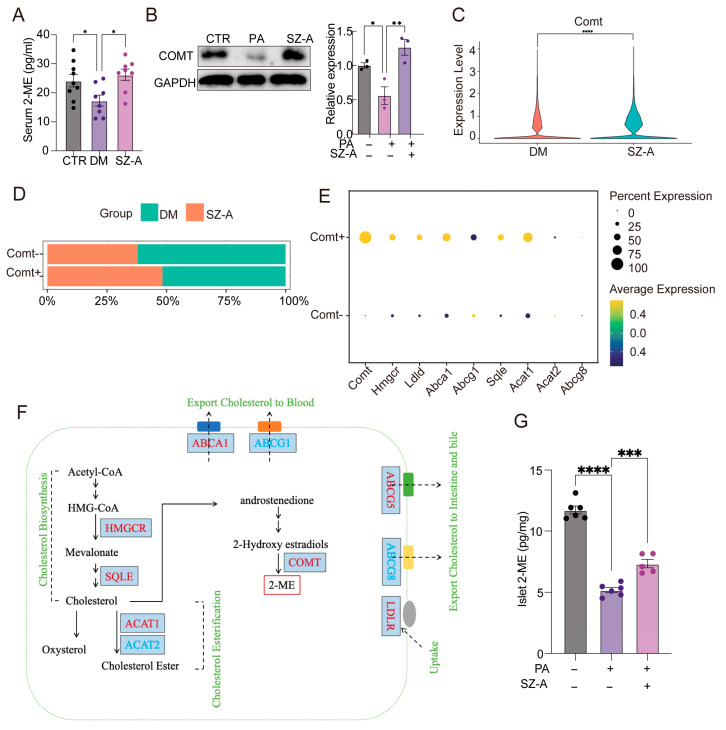
SZ-A elevate 2-ME levels by enhancing COMT expression in islets. (**A**) Serum 2-ME levels in CTR, DM, and those treated with SZ-A detected by ELISA (*n* = 8–9). (**B**) The expression of COMT protein in islets treated with CTR, 500 µmol/L PA, and 500 µmol/L PA combined with 50 µg/mL SZ-A. GAPDH served as a loading control (*n* = 3). (**C**) Violin plots of the relative expression of COMT genes in islets from DM and those treated with SZ-A. (**D**) The proportion of COMT^+^ and COMT^−^ subgroups in islets from DM and those treated with SZ-A. (**E**) Dot plots illustrating the relative expression levels of genes associated with cholesterol metabolism in islets from COMT^+^ and COMT^−^ subpopulations. (**F**) Schematic representation of the alterations in cholesterol-metabolism-related genes in COMT^+^ subpopulations. Genes are marked in red (upregulated) and blue (downregulated). (**G**) Islet 2-ME levels in CTR, 500 µmol/L PA, and 500 µmol/L PA combined with 50 µg/mL SZ-A detected by ELISA. The results assay was normalized to the protein concentration (*n* = 5–6). *, *p* < 0.05, **, *p* < 0.01, ***, *p* < 0.001, **** *p* < 0.0001.

To determine whether 2-ME reaches the pancreas through circulation, we measured its serum levels. The diabetic mice exhibited lower serum 2-ME levels than the controls, whereas SZ-A treatment significantly elevated these levels (*p* < 0.05, Figure 5A), indicating that SZ-A contribute to the restoration of systemic 2-ME levels.

COMT, a key enzyme in 2-ME biosynthesis, was significantly upregulated in primary islets following SZ-A treatment under PA-induced stress (*p* < 0.05, Figure 5B). To confirm this in vivo, we conducted an scRNA-seq analysis of islets from the diabetic and SZ-A-treated mice, revealing elevated COMT expression in the SZ-A group (Figure 5C). Based on COMT expression, islets were categorized into COMT^+^ and COMT^−^ subpopulations. The proportion of COMT^+^ cells was significantly increased in the SZ-A-treated diabetic mice (Figure 5D). Further gene expression analysis within the COMT^+^ subpopulation revealed a marked upregulation of key genes associated with cholesterol metabolism, including ABCA1, ACAT1, and ABCG5 (Figure 5E,F). These genes are critical for cholesterol transport, esterification, and homeostasis, potentially reflecting an increased capacity for cholesterol handling in these cells. Ultimately, direct quantification of 2-ME in islets confirmed a significant increase following SZ-A treatment (*p* < 0.05, Figure 5G), indicating enhanced intra-islet biosynthesis. These findings suggest that SZ-A promote the production of 2-ME through a dual mechanism: reshaping gut microbiota to influence systemic 2-ME levels and promoting 2-ME production within islets.

### 3.6. 2-ME Improves β-Cell Function by Inhibiting the Expression of Islet HIF-1α

**Figure 6:**
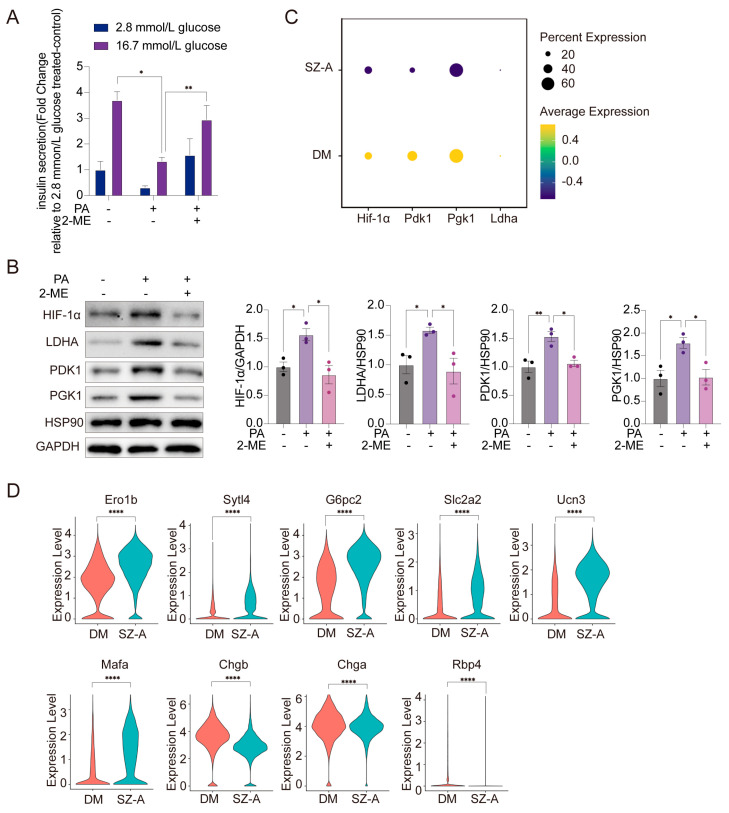
2-ME improves β-cell function by inhibiting the expression of islet HIF-1α. (**A**) GSIS in islets treated with CTR, 500 µmol/L PA, and 500 µmol/L PA combined with 500 nmol/L 2-ME (*n* = 3). (**B**) HIF-1α, LDHA, PGK1, and PDK1 protein expression levels in islets treated with CTR, 500 µmol/L PA, and 500 µmol/L PA combined with 500 nmol/L 2-ME. GAPDH served as a loading control for HIF-1α, PDK1, and PGK1, and Hsp90 served as a loading control for LDHA (*n* = 3). (**C**) Dot plots of the relative expression of HIF-1α, LDHA, PGK1, and PDK1 genes in islet from DM and those treated with SZ-A. (**D**) Violin plots of relative expression genes associated with β-cell maturation and function: Ero1lb, Sytl4, G6pc2, Slc2a2, Ucn3, and Mafa; and β-cell immuration: Chgb, Chga, and Rbp4. *, *p* < 0.05, **, *p* < 0.01, **** *p* < 0.0001.

To further assess the functional impact of 2-ME on insulin secretion, we measured GSIS in the primary islets of the mice treated with PA. Under high-glucose conditions, PA markedly impaired insulin secretion, whereas 2-ME treatment significantly restored GSIS in comparison to the PA-treated islets (*p* < 0.05). Notably, no significant differences in insulin secretion were observed between groups under low-glucose conditions (Figure 6A). These results suggest that 2-ME could effectively ameliorate PA-induced β-cell secretory dysfunction under high-glucose conditions.

2-ME has been well established as a potent inhibitor of HIF-1α [26], and sustained activation of HIF-1α has been shown to impair insulin secretion and contribute to β-cell dysfunction under diabetic conditions [27]. Given its role in HIF-1α inhibition, we evaluated the effects of 2-ME on HIF-1α signaling in primary islets exposed to PA. Our results demonstrated that PA stimulation increased HIF-1α protein expression, whereas treatment with 2-ME significantly reduced HIF-1α levels and suppressed the expression of its downstream target genes, including LDHA, PDK1, and PGK1 (*p* < 0.05) (Figure 6B). Given that our data demonstrate elevated levels of 2-ME following SZ-A treatment, it is plausible that SZ-A exert regulatory effects on HIF-1α signaling. Supporting this notion, scRNA-seq analysis revealed that islets from the SZ-A-treated mice displayed a reduction in HIF-1α expression and its downstream targets, specifically in β-cells, compared to those in the diabetic controls (Figure 6C). Additionally, SZ-A significantly upregulated key genes essential for β-cell maturation and function, including Mafa, Ucn3, G6pc2, Sytl4 and Ero1b, while downregulating immature β-cell markers such as Chga and Rbp4 (Figure 6D). These findings suggest that SZ-A promote β-cell maturation and insulin secretory function, potentially through 2-ME–mediated inhibition of HIF-1α signaling.

## 4. Discussion

SZ-A are recognized as safe and effective agents for diabetes management, with demonstrated potential to improve β-cell function and mass [13]. However, the precise mechanisms have yet to be fully elucidated. Previous studies using diabetic KKAy mice have shown that SZ-A modulate gut microbiota composition, increasing quantities of beneficial bacteria such as *Bacteroidaceae* and *Verrucomicrobia*, elevating fecal levels of acetic and propionic acids, and alleviating both systemic and intestinal inflammation [28]. These studies, however, focused primarily on microbiota compositional changes without further mechanistic exploration. In our study, we employed an HFD/STZ-induced diabetic mouse model, which presents distinct metabolic characteristics and a distinct gut microbiota composition relative to the KKAy model, likely influenced by environmental, dietary, and genetic differences. Given the observed alterations in lipid metabolism in this model, we further explored the relationship between gut microbiota and cholesterol metabolism. Emerging evidence has linked gut microbiota dysbiosis to impaired cholesterol metabolism. For instance, transplanting microbiota from individuals with elevated serum cholesterol into mice can induce a hypercholesterolemic phenotype [29], highlighting the role of microbiota in regulating host lipid homeostasis. Our results demonstrate that SZ-A treatment notably increased the abundance of bacteria involved in cholesterol metabolism, such as *Lactobacilli* and *the norank_f__Eubacterium_coprostanoligenes_group*. *Lactobacilli* can lower cholesterol via converting it into coprostanol, a process facilitated by their ability to produce cholesterol reductase [23]. Additionally, members of *the_Eubacterium_coprostanoligenes_group* possess the IsmA gene, which encodes an enzyme that specifically metabolizes cholesterol into coprostanol [24]. Our results reveal that *Lactobacillus* and *the norank_f__Eubacterium_coprostanoligenes_group* had a positive association with production of the cholesterol metabolite 2-ME. Furthermore, SZ-A treatment significantly increased the levels of various cholesterol metabolites, with the most notable increase observed for 2-ME.

Numerous studies have highlighted the potent anti-angiogenic, anti-inflammatory, and anti-tumor effects of 2-ME [30,31,32]. Beyond these well-known roles, 2-ME is increasingly recognized for its crucial function in glucose homeostasis, as evidenced by its ability to improve glucose tolerance, enhance GSIS, and promote β-cell mass expansion by stimulating β-cell proliferation in db/db mice [25]. Endogenous 2-ME is synthesized through the enzymatic action of COMT. Notably, its metabolic significance has been highlighted in studies on HFD-fed and pregnant mice, wherein reduced COMT activity was associated with impaired glucose tolerance. Conversely, 2-ME supplementation effectively alleviates these metabolic disturbances [19]. These findings underscore the critical role of COMT in regulating 2-ME production and maintaining glucose homeostasis. Building on this evidence, our study demonstrates that SZ-A treatment significantly increased the proportion of COMT^+^ cell subpopulations within the islets. This effect was accompanied by a marked upregulation of cholesterol-metabolism-related genes, particularly ABCA1, a key mediator of cholesterol transport and homeostasis. Notably, ABCA1 not only regulates cholesterol balance but also plays a crucial role in modulating insulin secretion in β-cells [33]. These findings suggest that SZ-A enhance endogenous 2-ME biosynthesis within islets and promote cholesterol metabolism, which may play a role in improving β-cell function.

2-ME is a well-established inhibitor of HIF-1α, known to suppress its protein synthesis, reduce hypoxia-induced DNA binding [34], and prevent its nuclear translocation [35]. In the context of T2DM, β-cells are subjected to both metabolic stress and hypoxia, conditions that contribute to β-cell dysfunction and are associated with elevated HIF-1α expression [36,37]. Notably, Ilegems et al. demonstrated that pharmacological inhibition of HIF-1α, including with PX-478, promotes the formation of mature insulin granules and improves β-cell function in diabetic mice [27]. Building on these findings, our study reveals that 2-ME effectively suppressed HIF-1α expression and downregulated its key downstream targets, which are known to drive a metabolic shift toward glycolysis under hypoxic conditions. By inhibiting this pathway, 2-ME may help alleviate metabolic stress in β-cells, ultimately supporting β-cell function and insulin secretion [38].

In this study, we highlight the multifaceted effects of SZ-A in diabetes treatment, identifying 2-ME as a crucial mediator of its protective effects on β-cells. While we have made progress in uncovering the mechanisms underlying SZ-A’s benefits, several limitations and unanswered questions remain. First, although correlation analysis linked *Lactobacillus* and *the norank_f__Eubacterium_coprostanoligenes_group* to elevated 2-ME levels, definitive evidence establishing a causal relationship between the microbiota and 2-ME is still lacking. Second, the enzymatic pathways governing the microbial conversion of cholesterol into 2-ME remain uncharacterized, particularly the relative contributions of bacterial cholesterol reductase versus host-derived modifications. Third, it remains unclear whether SZ-A promote β-cell function solely through 2-ME or if other protective microbial metabolites, such as short-chain fatty acids (SCFAs) and bile acids, also contribute to their therapeutic effects. Additionally, we observed significant COMT upregulation in islets, suggesting an enhanced local capacity for 2-ME production. However, it is still unknown whether β-cells possess the full complement of enzymatic machinery required to synthesize estrogenic precursors from cholesterol. Although the COMT^+^ β-cells exhibited increased expression of genes involved in cholesterol metabolism, this finding may merely reflect an improved capacity for lipid processing rather than active estrogen biosynthesis. It is therefore plausible that the substrate for COMT^−^mediated 2-ME synthesis is supplied systemically. Despite these uncertainties, our findings provide a foundational framework for understanding how SZ-A preserve β-cell function in diabetes by modulating gut microbial ecology and intra-islet metabolism.

## 5. Conclusions

Our study demonstrates that SZ-A can alleviate hyperglycemia in T2DM mice, partly by modulating the gut microbiota, notably via enriching the quantities of *Lactobacillus* and *the norank_f__Eubacterium_coprostanoligenes_group*, which are associated with increased 2-ME levels. Additionally, SZ-A increase the quantities of COMT^+^ cells in islets, suggesting intra-islet 2-ME production. Mechanistically, 2-ME enhances insulin secretion by suppressing HIF-1α signaling. These findings identify 2-ME as a key mediator linking the gut–islet axis and support its potential in restoring β-cell function.

## Data Availability

All data used in this study is available from the corresponding author upon reasonable request.

## Supplementary Materials

The following supporting information can be downloaded at: https://www.mdpi.com/article/10.3390/biomedicines13082013/s1, Figure S1: Chemical structures of DNJ, DAB, and FAG.
