# Supplementary figures and images for: Ramulus Mori (Sangzhi) Alkaloids Improve Pancreatic β-Cell Function Through Gut Microbial and Intra-Islet 2-Methoxyestradiol Biosynthesis
Source: Biomedicines. 2025 Aug 19;13(8):2013. doi: 10.3390/biomedicines13082013 (PMC12383358; doi:10.3390/biomedicines13082013)

## Supplementary materials

Figure S1. Chemical structures of DNJ, DAB, and FAG.

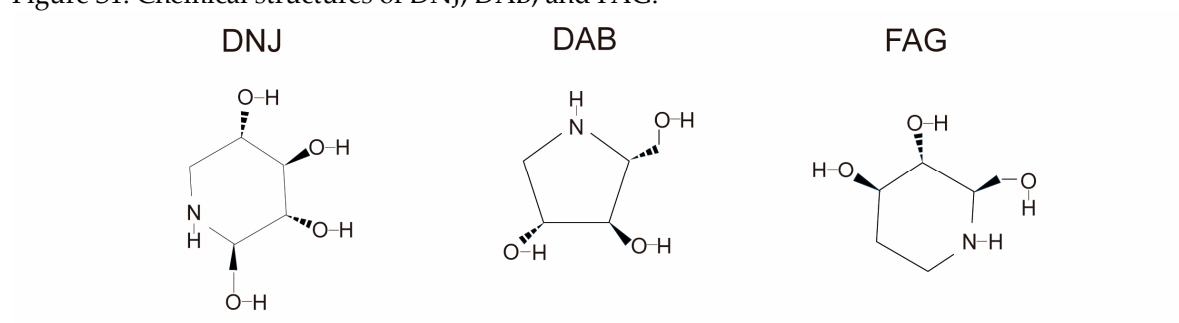

Supplement: Supplementary file 1 [file biomedicines-13-02013-s001.zip › biomedicines-3731017-supplementary.pdf]
